# Unflavored electronic cigarette exposure induces alterations in airway ciliary structure and function

**DOI:** 10.1186/s12931-025-03302-w

**Published:** 2025-07-02

**Authors:** Abdo Durra, Caroline Cherry, Coline Luo, Emily Hou, Andrew Frauenpreis, Arunima Purkayastha, Isabella Passamano, Sara Makanani, Kristen Castillo, Andrew Lund, Woosuk Choi, Chandani Sen, Rachana Chandran, Tammy Rickabaugh, Prashant Kaushal, Mehdi Bouhaddou, Eszter K. Vladar, Brigitte N. Gomperts

**Affiliations:** 1 Department of Pediatrics, David Geffen School of Medicine UCLA, UCLA Children’s Discovery and Innovation Institute, Mattel Children’s Hospital, Los Angeles, CA 90095 USA; 2 Department of Molecular and Medical Pharmacology, University of California, Los Angeles, CA 90095 USA; 3 Programs in Biomedical and Biological Sciences, University of Southern California Health Sciences, Los Angeles, CA 90033 USA; 4 Department of Microbiology, Immunology, and Molecular Genetics, University of California, Los Angeles, CA 90095 USA; 5 Institute for Quantitative and Computational Biosciences, University of California, Los Angeles, CA 90095 USA; 6 Medicine - Pulmonary Sciences & Critical Care Medicine, University of Colorado, Aurora, CO 80045 USA; 7 Jonsson Comprehensive Cancer Center, UCLA, Los Angeles, CA 90095 USA; 8 Eli and Edythe Broad Stem Cell Research Center, UCLA, Los Angeles, CA 90095 USA; 9 Division of Pulmonary and Critical Care Medicine, Department of Medicine, David Geffen School of Medicine, UCLA, Los Angeles, CA 90095 USA; 10 UCLA Environmental and Molecular Toxicology Interdepartmental Program, University of California, Los Angeles, CA 90095 USA

**Keywords:** Motile cilia, E-cigarettes, Ciliary beating frequency, Phosphoproteomics, Cytoskeletal remodeling, Rho GTPase

## Abstract

Electronic cigarettes (e-cigs) have been introduced as a safer alternative to traditional combustible cigarettes and have been growing in popularity. E-cig e-liquids all contain the carrier compounds, vegetable glycerin (VG), propylene glycol (PG), and nicotine, together with different flavors, but the effects of inhalation of these compounds on the airway are not well understood. This study investigates the effects of e-cig exposure on primary human airway epithelial cells grown in air–liquid interface (ALI) cultures, specifically focusing on mucociliary clearance, the lung’s primary host defense mechanism whereby pathogens and particles trapped by mucus are cleared by unidirectional beating by ciliated cells. We developed a microcontroller-based exposure system to reproducibly examine cellular and molecular changes in ALI cultures from e-cig exposure. Here we show heterogeneous, donor-dependent effects of different e-cig flavors on airway epithelial cells. Examining the effects of the unflavored carrier compounds common to all e-cigs, we found that ALI airway cultures exposed to PG:VG (30:70 ratio) with 5% nicotine unflavored e-cigs show a reduction in ciliary beat frequency. Moreover, using transmission electron microscopy, we identified defects in ciliary ultrastructure induced by unflavored e-cigs. Phosphoproteomic analysis uncovered changes in phosphorylation of proteins involved in cadherin and actin binding and the Rho GTPase signaling pathway, which are all involved in cytoskeletal remodeling that may influence ciliary structure and function. Altogether, our findings suggest that exposure to all e-cigs reduces mucociliary clearance.

**Supplementary Information:**

The online version contains supplementary material available at 10.1186/s12931-025-03302-w.

## Background

Combustible cigarettes have been known for decades to reduce mucociliary clearance (MCC) and are the primary driver of premalignant lesions and lung cancer [1]. In addition, they are one of the main factors associated with respiratory diseases such as Chronic Obstructive Pulmonary Disease (COPD), asthma, and idiopathic pulmonary fibrosis (IPF) [2–4]. During the recent decade, many alternatives to combustible cigarettes have emerged and e-cigarettes (e-cigs) were introduced as a safer alternative to combustible cigarettes and as a tool in smoking cessation. The base of the e-liquid in these e-cigs is comprised of Propylene Glycol (PG) and Vegetable Glycerin (VG) mixed at different ratios, to which flavoring compounds and nicotine are added. E-cig flavors can have a variety of flavor profiles, and they can be divided into multiple chemical classes depending on the functional groups that their chemical structures contain such as ketones, acetyls, aldehydes etc. [5]. Despite the short list of ingredients comprising e-liquids, chemical analyses using nontargeted methods have identified thousands of chemicals originating as contaminants from flavorings, e-cig components, e-liquid packaging, or are formed by thermal decomposition of the e-liquid solvents over time [6]. Furthermore, the heating process of the coil inside e-cigs produces toxic metals that end up in e-liquids and aerosols [7, 8]. Moreover, the rising popularity of single-use e-cigs and the lack of proper avenues to dispose and recycle their batteries contribute to environmental issues because of nicotine and lithium leaching into water and soil [9].

The lungs are exposed to particles, toxins and pathogens from the environment with every breath. The airway epithelium lining the cartilaginous airways helps to protect the lungs from these exposures creating a chemical and physical barrier to limit the passage of inhaled foreign matter. This first line of defense continuously clears the airway by MCC [10, 11]. MCC consists of two interdependent components: 1) hair-like motile cilia on ciliated cells that beat unidirectionally in a synchronous manner and 2) the mucus and serous secretions from the secretory cells and submucosal glands that entrap pathogens and debris [12–14]. The synergistic function of these two components creates a host defense system for the lungs. As a result, defects in either of these components compromise the function of MCC and lead to the pathogenesis of chronic pulmonary disorders.

E-cig use has also been shown to negatively impact lung health. Studies show e-cig users experience an increase in airway resistance, making it more difficult for them to breathe [15]. E-cigs have also been shown to induce lung inflammation comparable to that seen in asthma or bronchitis [16]. E-cigs use also leads to an increased susceptibility to infections which stems from damage to the airway barrier function [17]. Combustible cigarettes have been shown to induce physiological alterations in ciliary function and activity, morphological alterations of cilia, and decreased mucociliary clearance [18–24]. Similar to combustible cigarettes, there have been reports linking e-cig use and dysregulated MCC. Various studies using cell models have described how e-cigs induce a decrease in cell viability and ion transport and an increase in mucus viscosity [25–27]. Animal studies have found e-cig exposure also decreases cell viability and increases mucus production [28–30]. One study in sheep found e-cig aerosols to reduce tracheal mucus velocity, which is a measurement of mucociliary clearance function [27]. Many of these studies highlight the effect of e-cigs on the mucosal component of MCC. The focus of our study is on the ciliary component and the effects of e-cig exposure on motile cilia beat frequency (CBF) and cilia motility. To understand the potential effects of e-cigs on cilia, it is important to first understand the normal structure and function of cilia. Proper ciliary structure is crucial for effective ciliary beat frequency, and MCC.

The primary ultrastructure of the cilium is an axoneme which is composed of a central pair of microtubules that is surrounded by nine outer microtubule doublets (known as 9 + 2) that are bound to the center by radial spoke proteins [31]. The doublets have dynein arms which are molecular motors that enable ciliary beating. There are many instances of abnormal or disorganized axonemes characterized by separated central pair or missing central pair with an outer doublet “falling” into the center of the axoneme to replace the central pair. These ultrastructural defects are used to diagnose the genetic disorder, primary ciliary dyskinesia (PCD) [32]. PCD is caused by mutations in genes that affect ciliary structures. Patients with PCD have dysregulated MCC and abuildup of mucus in their airways.

In this study, we developed an *in-vitro* exposure system and demonstrated that short and low-level e-cig exposures with different flavors have a heterogeneous effect on airway epithelial cell types grown in air–liquid interface (ALI) cultures. Moreover, different donors responded differently to the different flavors. This heterogeneity in response to flavors led us to study unflavored e-cigs, which contain the components that are present in all e-cigs. We showed that unflavored e-cigs alter the function of ciliated cells and reduce their beat frequency. Transmission electron microscopy revealed ciliary ultrastructural changes from airway ALI exposures to unflavored e-cigs. We then performed multi-omics analyses and found minimal significant changes at the transcript and protein levels after ALI exposure to unflavored e-cigs. However, we identified significant changes in post-translational modifications (PTMs) involving protein phosphorylation. Further analysis identified possible correlations between these PTM alterations and ciliary dysfunction, revealing that acute and low-level exposures to e-cigs can have detrimental effects on MCC, leading to poor airway health.

## Methods

### Human tissue procurement

Large airways and bronchial tissues were acquired from de-identified normal human lung donors after lung transplantations at the Ronald Reagan UCLA Medical Center with no prior history of smoking or lung disease. In addition, non-transplantable donor research lungs were obtained from the International Institute for the Advancement of Medicine (IIAM). Deidentified human lung tissue was procured under an Institutional Review Board-approved protocol at the David Geffen School of Medicine at UCLA (exemption# 21–000390).

### Airway basal stem cell (ABSC) isolation

Human ABSCs were isolated following a previously published method by our laboratory [33–36]. Briefly, tracheas were dissected, cleaned, and incubated in 16U/mL dispase for 45 min at room temperature. Tracheas were then incubated in 0.5 mg/mL DNase for another 45 min at room temperature. The epithelium was stripped under a dissecting microscope and incubated in 0.1% Trypsin–EDTA for 30 min shaking at 37 °C to generate a single-cell suspension. Isolated cells were passed through a 40 μm strainer and cryopreserved using Bambanker freezing media (FUJIFILM Wako Chemicals USA). ABSCs from two to three donors were used for all experiments.

### Air–Liquid Interface (ALI) cultures

24-well, 6.5 mm transwells with 0.4 μm pore polyester membrane inserts were coated with 0.5 mg/mL collagen type IV from human placenta dissolved in acetic acid and diluted in cell grade water at a ratio of 1:10. 100 μl was added to each transwell and allowed to air dry. ABSCs were seeded at 100,000 cells per well directly onto collagen-coated transwells and allowed to grow in the submerged phase of culture for 4–5 days with 500 μL in the basal chamber and 200 μL in the apical chamber of PneumaCult™ Ex- Plus medium (StemCell Technologies #05040). ALI cultures were then established and cultured with only 500 μL PneumaCult™ ALI Maintenance medium (StemCell Technologies #05001) in the basal chamber. Media was changed every other day and cultures were maintained in humidified incubator at 37 °C with 5% CO_2_. Cultures are considered mature and differentiated after 21 days since initiating ALI.

### E-cigarette exposure system

Airway cells grown in ALI were exposed to e-cigarette aerosols for 5 min 2X a day for 10 consecutive days using a 3-L air-tight chamber equipped with an inlet and an outlet (Kent Scientific). An air pump (Adafruit Industries) is linked to the inlet on one end and to a Juul e-cigarette device with a refillable pod (BLANKZ) on the other using an adapter. The other inlet is left disconnected and open during the pump operation to avoid pressure buildup. The air pump is controlled by an Arduino UNO microcontroller which is fitted with a relay shield mod (Seeed Technology Co). The Arduino is programmed to activate the air pump 3 times, each puff lasting 3 s with an inter-puff duration of 6 s. Following the puffing, the chamber’s outlet is closed. The entire exposure process lasts 5 min.

### E-liquid solution

The e-liquid solution for unflavored e-cigs was made using propylene glycol, glycerol, and nicotine obtained from Sigma-Aldrich (St. Louis, MO, USA) Cells were exposed to unflavored e-cigs comprised of a PG:VG (30:70 ratio) with 5% nicotine. The e-liquid was placed in an empty 1 ml JUUL pod (BLANKZ).

### Antibodies

Antibodies used in immunofluorescence analysis were anti-Keratin 5 antibody (Cat# 905,501, BioLegend, San Diego, CA, USA), anti-acetylated Tubulin antibody (Cat# T7451, Millipore Sigma, Burlington, MA, USA), and anti-Muc5AC antibody (Cat# MA5-12,178, Invitrogen, Carlsbad, CA, USA)

### High-speed video microscopy

Using a previously published protocol, we acquired high-speed microscopy videos [37]. On the day of collection, the transmembrane of ALI transwells was cut and mounted on a glass microscopy slide with #1.5 coverslip. We used a Zeiss Axiom Observer 7 fully motorized research inverted microscope with a motorized scanning stage with a plan-apo 63 × oil immersion 1.4 NA objective and a Hamamatsu Flash 4.0 version 3 camera. Utilizing this setup, the entire height of the epithelium and cilia was visible to be captured. We used Zen 3.3 software to acquire DIC videos for 3 s at 400 frames/sec. We acquired videos from twenty fields of view (FOVs) per ALI membrane in a predetermined unbiased layout in the shape of a plus sign. This approach captures data from both central and peripheral regions, minimizing selection bias. Representative videos are in the supplementary material of the manuscript.

### Immunocytochemistry

Human ALI cultures were fixed in 4% paraformaldehyde for 15 min followed by permeabilization with 0.5% Triton-X in Tris-Buffered Saline for 10 min. Cells were then blocked using serum-free protein block (Dako X090930) for one hour at room temperature and incubated with primary antibodies overnight at 4 °C. After 4 washes with Tris-Buffered Saline and Tween-20 (TBST), the primary antibodies were probed with fluorescence-conjugated secondary antibodies for 1 h in darkness. After probing, the cells were washed and mounted using Vectashield Vibrance® Antifade Mounting Medium (Vector Labs H-1700). Immunofluorescence images were obtained using an or LSM880 Zeiss confocal microscope and composite images were generated using Fiji.

### Transmission electron microscopy

The tissues were fixed in 2% glutaraldehyde, and 4% paraformaldehyde in 0.1 M NaCacodylate buffer, pH 7.4 for 15 min at room temperature, then changed to fresh fixative and left at 4 °C overnight. Samples were osmicated, stained with uranyl acetate, then dehydrated with a graded ethanol series. Filters were cut out of the plastic supports, then infiltrated with EMBed 812 (Electron Microscopy Sciences). 80 nm sections were mounted onto copper grids and analyzed with a JEOL JEM-1400plusTransmission Electron Microscope (JEOL).

All other details are described in the extended Methods section of the data supplement.

## Results

### Human air liquid interface cultures exposed to e-cigarette aerosols with different flavors display donor heterogeneity

**Fig. 1:**
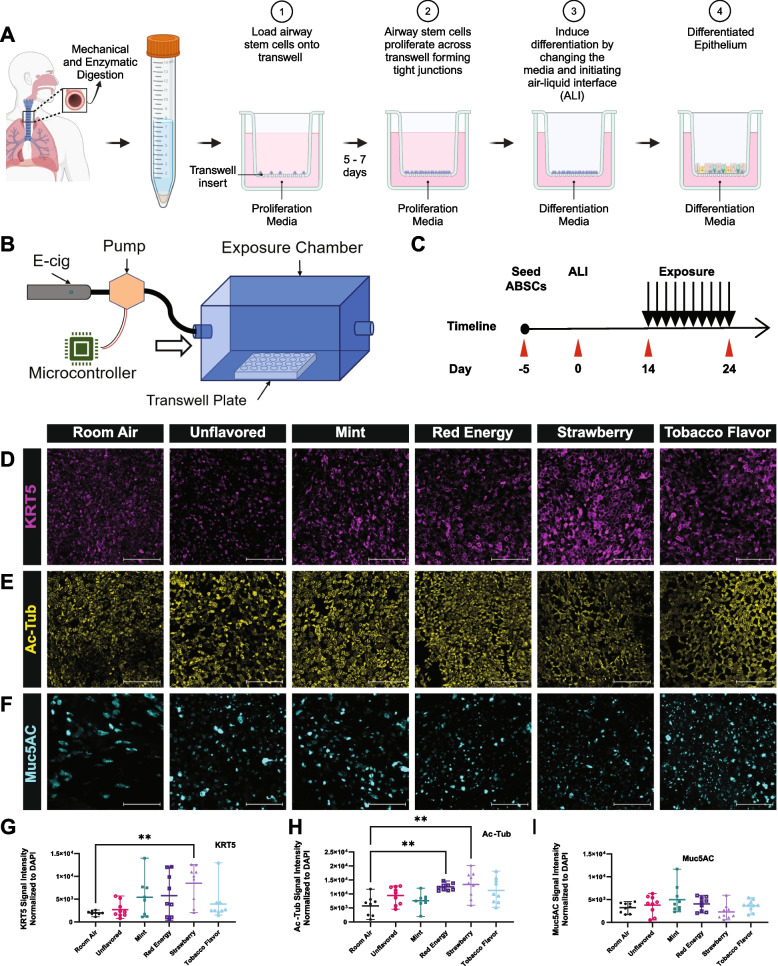
Donor and flavor-specific responses to e-cig aerosols in human bronchial epithelial cells grown in ALI. **A** Schematic illustrating ABSC isolation from donor tissue, seeding into tissue culture transwell inserts and initiating air–liquid interface (ALI). **B** Schematic showing basic components of the exposure system. **C** Schematic outlining the timeline of ABSC seeding and ALI initiation followed by e-cig aerosol exposure. **D** Representative IF images of Keratin 5 (KRT5)-expressing (magenta) ABSCs in ALI cultures across the six exposure conditions of room air control, unflavored PG:VG (30:70 ratio) with 5% nicotine, and mint flavored, red energy flavored, strawberry flavored, and tobacco flavored e-cigs. **E** Representative IF images of acetylated alpha tubulin (Ac-Tub) (yellow) ciliated cells in ALI cultures across the six exposure conditions. **F** Representative IF images of Muc5AC (cyan) mucus cells in ALI cultures across the six exposure conditions. **G** Quantification of number of Keratin 5 + across the six exposure groups. **H** Quantification of number of Ac-Tub + across the six exposure groups. **I** Quantification of number of Muc5AC + mucus cells across the six exposure groups. Graphs represent mean ± SEM, *n* = 3 biological replicates (3 different donors), each with 3 technical replicates (3 ALI transwell cultures derived from each of the 3 different donors) for a total of 9 replicates per exposure condition. Each dot represents a replicate. *P* values are calculated from all technical replicates across the biological replicates as described above. Scale bar = 100 μm

We isolated human airway basal stem cells (ABSCs) from bronchial tissue from three healthy donors. These ABSCs were cultured under submerged conditions in proliferation media for 5 days, before air-liquid interface (ALI) conditions were initiated using differentiation media, as previously described (Fig. 1 A) [38–40].

We devised an exposure system to perform e-cig aerosol exposures on ALI cultures (Fig. 1 B). We examined four popular e-cig flavors that are sold at local smoke shops to demonstrate their effects on the bronchial mucociliary epithelium. Aerosol treatments started on day 14 post initiation of ALI and continued to day 24 (Fig. 1 C). Although full differentiation of ALI cultures typically occurs around day 21, we initiated exposures at day 14 to model a state of ongoing epithelial differentiation, which mimics airway epithelial repair following injury. This timing allowed us to examine the effects of e-cig aerosol exposure on both regenerating and differentiated surface epithelium. We optimized our aerosol exposure time and duration to obtain a dose that allowed us to examine the effects of e-cig aerosol exposure on ABSCs and mucociliary cells with minimal cell death (data not shown). Our optimized aerosol exposures consisted of three e-cig puffs of 3 s each, with a 5-min exposure time, twice a day for 10 days.

To study the effects of e-cig flavors on the mucociliary epithelium, we assessed four flavors: mint, red energy, strawberry, and tobacco, all of which had PG:VG (30:70 ratio) with 5% nicotine content. These e-cig products were compared to an unflavored e-cig group comprised of a PG:VG (30:70 ratio) with 5% nicotine and room air exposure as a control. We performed the 20 exposures over 10 days to mimic an acute exposure in the airway. We then isolated the ALI membranes, and using immunocytochemistry, we examined changes in airway epithelial cell subpopulation in different exposure conditions. We focused our analysis on three cell types of the airway epithelium, keratin 5 (KRT5) expressing ABSCs, acetylated-alpha tubulin (Ac-Tub) expressing ciliated cells and mucin 5AC (Muc5AC) expressing goblet cells. Minimal changes in ABSC and mucociliary cell populations were observed following the exposure of e-cig aerosols from these four flavors when compared to the unflavored group and room air control. When quantifying the data across all donors and comparing the effects of e-cigs to the room air exposure, we found a large amount of heterogeneity in cell response and therefore only found a significant increase in the number of basal cells after the strawberry-flavored exposure (Fig. 1 D, G), and an increase in ciliated cells after both the Red Energy and Strawberry flavored exposures (Fig. 1 E, H), whereas there were no significant changes in goblet cells (Fig. 1 F, I).

Given the known heterogeneity of people’s clinical responses to e-cigs [41, 42], we next examined individual donors and identified heterogeneity in the changes in airway epithelial cell types in response to e-cig flavors across donors (Fig. 1S A-C). In general, donors 2 and 3 had a significantly increased response to all flavors in the three cell types compared to donor 1 (Fig. 1S A-C). Donors 2 and 3 had a significantly higher response in KRT5 basal cells across all flavored exposures compared to donor 1 (Fig. 1S A). Donors 2 and 3 also had a significantly higher response than donor 1 in Ac-Tub + ciliated cells in the Strawberry and Tobacco flavors, and donor 2 had a higher response than donor 1 in the unflavored e-cig exposure (Fig. 1S B). In Muc5AC cells, donor 3 had significant changes compared to donor 2 in strawberry and red energy exposures, and significant changes compared to donors 1 and 2 in mint and unflavored exposures (Fig. 1S C). The flavor-dependent variability seen across the three donors is expected as the chemical composition of these synthetic flavors and the additives used by their manufacturers can vary greatly. We also saw donor-to-donor heterogeneity in response to different e-cig flavors across cell types (Fig. 1S D-F). Expression of KRT5 basal cells had no significant changes in response across all flavors in donor 1; however, only strawberry flavor had a significant change in donor 2, and all flavors in donor 3 had a significant change (Fig. 1S D). Response in ciliated cells also varied across donors where donor 1 had an increase in red energy flavor, donor 2 in unflavored, red energy, strawberry and tobacco flavors, and donor 3 only in strawberry flavors (Fig. 1S E). Finally, the expression of Muc5AC had a significant response only in donor 3 when exposed to mint flavor (Fig. 1S F).

These findings highlight the considerable challenge in studying the effects of e-cigs due to the variability across flavors. To address this complexity, we decided in our subsequent studies to investigate the effects of the shared base components of e-liquids: PG, VG, and nicotine on bronchial epithelial cells. Using this approach, we hypothesized that we would be able to infer airway cellular changes that would potentially occur from exposure to all types of e-cigs.

### Unflavored e-cig aerosols induce reduction in the relative ciliary beat frequency of ciliated cells and disruption of ciliary ultrastructure

To assess the effects of unflavored e-cig aerosols on ALI cultures, we differentiated ABSCs from two additional healthy donors in ALI cultures, separate to those donors assessed in Fig. 1 and exposed them to unflavored e-cigs comprised of a PG:VG (30:70 ratio) with 5% nicotine (Fig. 1 A, B). We performed the exposures for 5 min twice per day for 10 days for a representative acute airway exposure (Fig. 1C) and after the last exposure on the last day, we isolated the ALI membranes and acquired high speed microscopy videos (Supplemental Video 1 shows cilia motility with room air exposure and Supplemental Video 2 shows cilia motility after PG:VG (30:70 ratio) with 5% nicotine exposure). Then we utilized a MATLAB analysis pipeline to characterize ciliary activity across all videos in the unflavored e-cig group compared to the room air control in both donors [37].

**Fig. 2:**
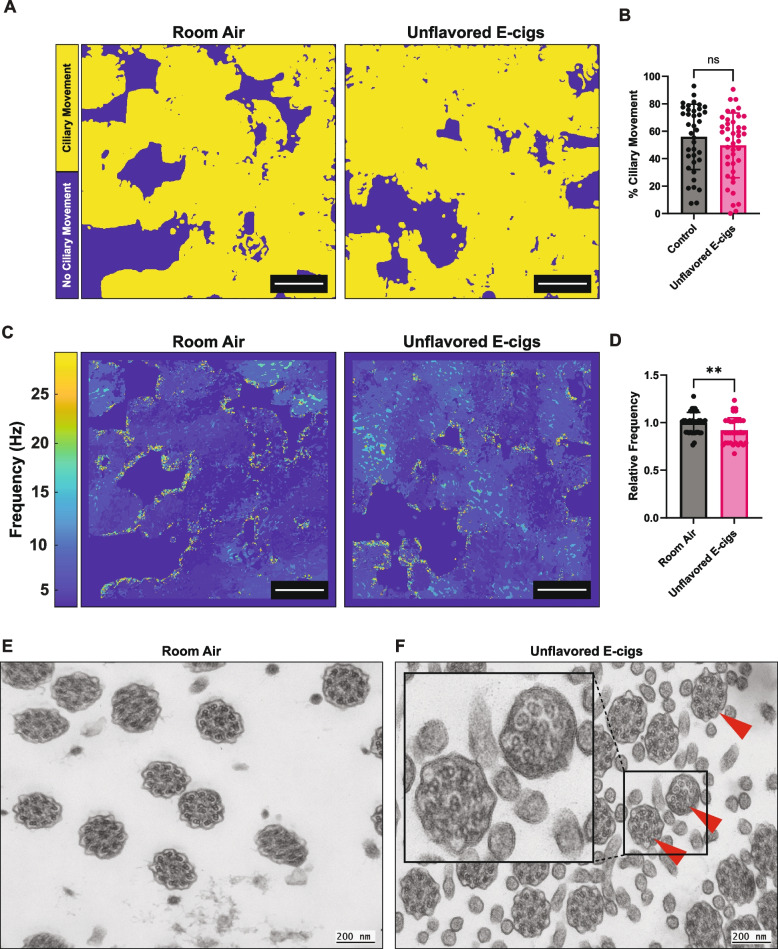
Unflavored e-cig aerosols induce reduction in the relative beat frequency of ciliated cells and disruption of ciliary ultrastructure. **A** Representative movement maps of field of views (FOVs) from microscopy videos of ALI cultures exposed to unflavored e-cig aerosols or air control. **B** Quantification of percent ciliary movements from microscopy videos of ALI cultures exposed to unflavored e-cig aerosols or air control. **C** Representative ciliary beat frequency (CBF) maps of FOVs from microscopy videos of ALI cultures exposed to unflavored e-cig aerosols or air control. **D** Quantification of average CBF from microscopy videos of ALI cultures exposed to unflavored e-cig aerosols or air control. **E** *p* = 0.0153 (*n* = 2 biological replicates, 20 technical replicates). Each dot represents one video per condition. TEM images of cilia axonemes from ALI cultures that were exposed to unflavored e-cig aerosols or air control. Scale bar 200 nm. (*n* = 1 biological replicate, 2 technical replicates) Red arrows point to ultrastructural defects

Our data shows the unflavored e-cig exposure did not induce a significant change in motile cilium coverage compared to room air control and there was no significant difference across the two patients tested (Fig. 2 A, B, Fig. 2S A). This correlated with the immunocytochemistry data for Ac-Tub (Fig. 1 E, H). However, the unflavored e-cig exposures reduced the motile CBF compared to the room air control (Fig. 2 C, D, Fig. 2S B). This indicates that the e-cig exposure did not cause loss of ciliated cells and the percentage of cilia capable of movement remains the same, but e-cig exposure is impacting the function of the cilia, and they are beating at a slower rate. This could be due to structural damage or alterations in the molecular machinery that drives ciliary movement. To assess ciliary ultrastructure we performed transmission electron microscopy (TEM) of room air control and unflavored e-cig exposed ALI cultures (Fig. 2 E, F) and observed defects in the 9 + 2 arrangement of ciliary axonemal microtubules in 24% of the e-cig exposed axonemes compared to 13% in the room air exposed axonemes [31]. These included loss or displacement of the central pair of microtubules and reduction in the number of outer microtubule doublets, defects reminiscent of primary ciliary dyskinesia (PCD), a genetic disorder characterized by abnormal mucociliary clearance. We hypothesized that these abnormalities in ciliary ultrastructure were due to an e-cig induced change in transcription, translation or post-translational modifications of ciliary proteins.

### Unflavored e-cig aerosols induce alterations in the phosphoproteomic profile of bronchial epithelial cells grown in ALI cultures

**Fig. 3:**
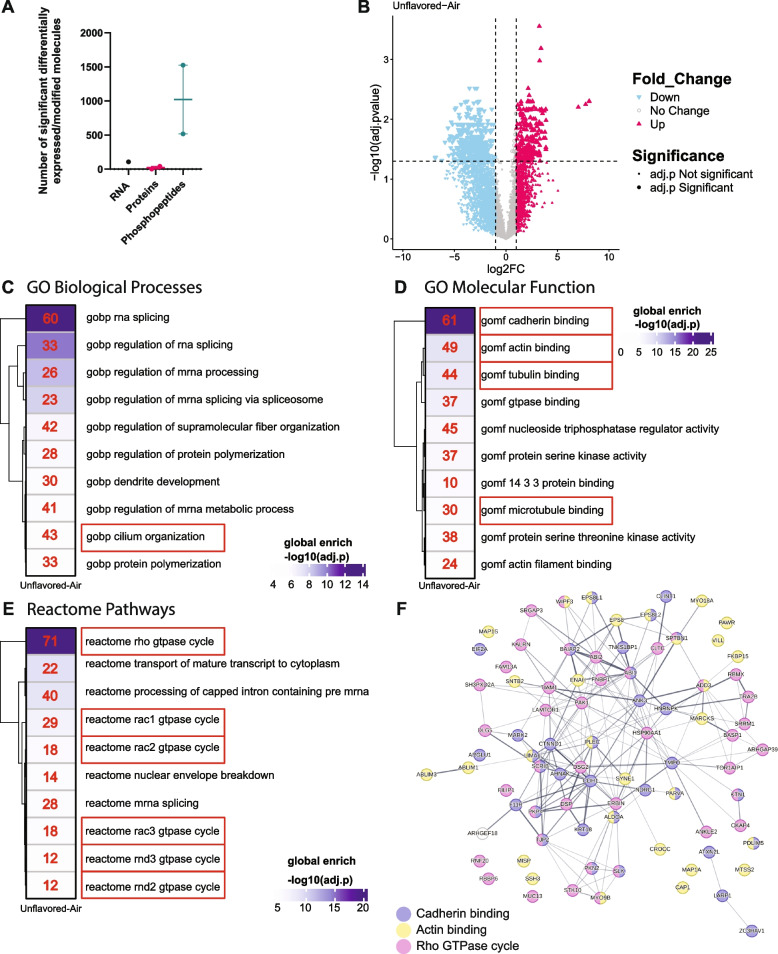
Unflavored e-cigarette aerosols induce alterations in the phosphoproteomic profile of bronchial epithelial cells grown in ALI. **A** Comparison of significantly altered molecules in airway cells following exposure to unflavored e-cig aerosols. The number of differentially expressed genes (RNA), proteins, and phosphopeptides are shown. (RNA: *n* = 2 biological replicates, 2 technical replicates. Protein; *n* = 2 biological replicate, 3 technical replicates, Phosphopeptides: *n* = 1 biological replicate, 3 technical replicates) A molecule was considered significantly altered if it exhibited a fold change > 1 or < −1 and an adjusted *p*-value ≤ 0.05. **B** Volcano plots depicting differential phosphorylation of phosphorylated sites in airway cells following exposure to unflavored e-cig aerosols compared to room air control. Each point represents a unique phosphorylation site. The x-axis shows the log2 fold change (log2FC) in phosphorylation, while the y-axis shows the -log10 of the adjusted p-value. Cyan points indicate decreased phosphorylation (log_2_ fold change <—1), magenta points indicate increased phosphorylation (log_2_ fold change > 1), and grey points indicate phosphorylation sites that did not meet those criteria. Point size corresponds to significance, with larger points representing phosphorylation sites with an adjusted p-value ≤ 0.05. **C**-**E** Heatmaps depicting pathway analysis for the proteins with dysregulated phosphorylation that belong to Gene Ontology (GO) Biological Processes terms (**C**), GO Molecular Function ontology (**D**), and Reactome Pathways Database (**E**). For all heatmaps, the color intensity of each box represents the degree of enrichment for a specific GO term calculated as -log10(adjusted *p*-value). A gradient from white (low enrichment) to purple (high enrichment) indicates the strength of the association. Numbers within each box show the number of genes from the input list associated with that term that have statistically significant enrichment (*p*-value < 0.05). Red boxes highlight terms of interest. (H) STRING network of the enriched proteins belonging to the GO terms “gomf cadherin binding” (purple) and “gomf actin binding” (yellow) and REACTOM term “rho GTPase cycle” (pink) were analyzed using STRING interaction network analysis. Interaction confidence was displayed as the edge thickness. Medium confidence interactions (combined score > 0.4) were used for the analysis. Phosphopeptides: *n* = 1 biological replicate, 3 technical replicates)

While the functional and structural effects of unflavored e-cigs on ciliated cells were apparent (Fig. 2), the molecular mechanisms driving these alterations remained unclear. To explore this, we performed a comprehensive analysis of the cellular response following the unflavored e-cig exposure. We cultured and differentiated ABSCs in ALI and exposed them to unflavored e-cigs according to our exposure regimen (Fig. 1C). Our comprehensive analysis included global RNA sequencing to assess transcriptional changes, mass spectrometry protein abundance measurements to evaluate proteomic alterations, and mass spectrometry phosphoproteomics profiling to identify changes in post-translational modifications (PTMs), specifically, phosphorylation. Surprisingly, following our regimen of 5-min exposure time, twice a day for 10 days, we observed very few significant changes in gene expression (Fig. 2S C, D) and there were very few proteins that were significantly differentially expressed in comparison to our room air control (Fig. 2S E). Conversely, we observed striking remodeling at the level of PTMs, specifically phosphorylation (Fig. 3A, Fig. 2S F–H, Supplementary Table 1). This poor correlation between protein phosphorylation and protein or mRNA abundance suggests that phosphorylation is driven independently from changes in protein abundance. We identified 271 up and 1255 down differentially phosphorylated sites as shown in the volcano plot (Fig. 3B).

Recognizing that phosphorylation can occur at multiple sites within a protein, we collapsed our analysis to the protein level, considering all proteins showing significant changes in phosphorylation at any site. By doing so, we aimed to delineate the pathways regulated by phosphorylation. Following this method, we identified all proteins with phosphorylation sites that decreased following e-cig exposure (relative to room air): 658 proteins had decreased phosphorylation. Conversely, we identified 108 proteins with increased phosphorylation following e-cig exposure.

We next examined those proteins with dysregulated phosphorylation and performed pathway analysis using bioinformatics [43]. The analysis using gene ontology (GO) Biological Processes ontology identified a significant enrichment of the term “cilium organization” (Fig. 3C, Supplementary Table 2). GO Molecular Function ontology analysis identified significant enrichment in terms related to “gomf cadherin binding” and “gomf actin binding”, in addition to “tubulin binding” and “microtubule binding” (Fig. 3D, Supplementary Table 2). Reactome pathways revealed enrichment in many terms belonging to the Rho GTPase signaling pathway (Fig. 3E, Supplementary Table 2). Interestingly, these terms are involved in the regulation of cilium organization [44]. Both cadherins and actin binding play crucial roles in the differentiation of ciliated cells in the airway [45, 46].

Next, we took all the proteins with significantly dysregulated phosphorylation (in either direction) and that were also in the terms cadherin and actin binding, along with rho gtpase, and created a network in STRING to show their connectivity (Fig. 3 F). The network revealed a large degree of interconnectedness between proteins, indicating interrelatedness of these pathways. Furthermore, these pathways have all been linked to ciliogenesis [47] and we hypothesize their dysregulation by e-cig exposure contributes to the ciliary dysfunction we observed.

## Discussion

The proliferation of e-cig products and flavors over the past decade presents a significant challenge in understanding their health effects. With over 15,000 flavors documented in online sales [48], selection of representative products for research is challenging. We saw this in our studies with a heterogeneity of response to flavors and heterogeneity between the donors we examined. These data are not surprising given the large difference in the chemical composition of e-cig flavors and how different people respond differently to environmental exposures.

However, a reproducible, consistent finding when we exposed ALI cultures to unflavored e-cigs consisting of PG:VG (30:70 ratio) with 5% nicotine is a reduction in ciliary beat frequency. This was validated by identifying ultrastructural defects after e-cig exposure. Furthermore, we show that the most significant molecular changes after unflavored e-cig exposure are post-translational modifications in the form of differential phosphorylation of specific proteins involved in the cytoskeleton, cell–cell adhesion and signaling pathways involved in cilium organization.

E-cigs use patterns vary significantly among users, with reported daily puff counts ranging from 15 to 1000 [49]. This variability poses a challenge for designing *in-vitro* exposure regimens that accurately reflect real-world usage. In this study, we opted for an exposure protocol of 20 exposures over 10 days each lasting 5 min. This exposure regimen, while substantially lower than reported e-cig use, served as an effective starting point and elicited a reproducible decrease in cilia beat frequency from all ALI cultures. Future studies utilizing ALI cultures could explore more intense exposure regimens by increasing the number and/or duration of exposures to potentially amplify cellular responses. However, it is crucial to consider that ALI cultures lack the complex immune components necessary to resolve inflammation. Therefore, careful optimization of the exposure parameters is necessary to avoid overwhelming the response capacity of ALI cultures.

When assessing the effects of e-cigs exposure, we investigated their effect on the three primary cell types of the airway epithelium: basal stem cells, ciliated cells, and goblet cells. However, upon a closer examination of individual ABSC donor responses, we uncovered significant variability. Particularly, cells from donor 1 on average exhibited a less profound response to the e-cig exposures across the different flavors compared to the other donors, while cells from donor 3 displayed on average a heightened response. This donor-to-donor heterogeneity likely stems from a combination of factors, including genetic predisposition, lifestyle differences, and preexisting conditions. These findings portray the importance of considering donor variability in response to e-cig exposure. Future studies are needed to capture a wider range of responses.

To assess the impact of e-cig exposure on the ciliary component of MCC, we evaluated its effects on motile cilia beating frequency (CBF), density, and distribution. Analyzing ciliary activity requires specialized tools to measure CBF from microscopy videos. Many of them only measure CBF and predominately in biased user selected areas [50–52]. To measure CBF and capture a comprehensive assessment of motile cilia coverage, we followed a previously published protocol that removes human bias and enables high throughput analysis of hundreds of videos in a short amount of time. One limitation of this protocol is that it only detects motile cilia requiring other assays to fully characterize total cilia coverage including the non-motile ones. Our data show that while the motile cilia coverage is not significantly affected following e-cig exposure, the motile cilia have a lower CBF due to the exposure. In addition, we also evaluated the ultrastructure of cilia using TEM and identified defects in the ciliary axonemal microtubules in ALI cultures exposed to unflavored e-cigs aerosols.

Cellular responses to stimuli operate at various levels and time scales. While RNA and protein levels are relatively stable, post-translational modifications (PTMs), such as phosphorylation, offer a rapid mechanism of cellular responsiveness. Phosphorylation enables cells to adapt quickly to stimuli without necessitating changes in protein levels. In the context of our experiments, where airway cells were acutely exposed to e-cig aerosols, it is plausible that the observed changes in the phosphoproteomic profile reflected this rapid adaptation mechanism. The significantly greater response at the PTM level compared to RNA and protein levels suggests that phosphorylation plays a crucial role in the immediate cellular response to e-cig exposure. Furthermore, the lack of significant changes in protein abundance, despite our 10-day exposure regimen, suggests the remodeling of the phosphoproteome may be transient. This highlights the importance of investigating PTMs to gain a comprehensive understanding of the cellular effects of e-cigs.

Our phosphoproteomics data elucidates the differential phosphorylation of proteins involved in both cadherin and actin binding as the common response to unflavored e-cig exposure compared to room air control. Cadherins, specifically E-cadherin, are transmembrane proteins that mediate cell–cell adhesion and regulate signaling pathways involved in ciliogenesis [53]. Planar cell polarity (PCP) is essential for the beating of motile cilia and cadherins are required for establishing PCP in ciliated cells [54]. Meanwhile, actin filaments form a dynamic network within cells, providing structural support and facilitating the anchoring of basal bodies, which are the microtubule-based structures that give rise to cilia [46]. Moreover, RhoA-mediated apical actin enrichment has been shown to be required for ciliogenesis [47]. The Rho GTPase signaling pathway was another term that was significantly enriched based on the pathway analyses. Therefore, dysregulation of the Rho GTPase signaling and disruption of actin binding can affect the formation and stability of the apical actin web, which is crucial for anchoring basal bodies and ensuring the proper positioning and orientation of cilia and could explain the alterations in ultrastructure seen previously.

Among the limitations of this study, we observed notable variability among human donors, which is expected given the biological diversity in airway epithelial cell populations, albeit all patients were presumed healthy with no history of cigarette use or prior lung disease. This donor variability complicates the interpretation of consistent trends and responses to vaping exposure. Furthermore, our sample size was limited due to the logistical challenges associated with obtaining primary human airway tissue, which may reduce the generalizability of our findings. While in vitro exposure models using differentiated human airway epithelial cells offer a controlled and reproducible system, they may not fully recapitulate the complex and dynamic nature of in vivo exposures. Factors such as immune system interactions, tissue remodeling, and chronic exposure to unflavored e-cig aerosols are not captured in this current model. Finally, in this study we used a standardized base consisting of propylene glycol, vegetable glycerin and nicotine, which is common in most e-liquids; however, this does not account for the additional chemical variability introduced by different flavoring agents. These limitations highlight the need for further studies incorporating larger donor cohorts, longer exposure durations, and complementary in vivo models to better understand the physiological impact of vaping on human airway health. Nonetheless, the robust effects on protein phosphorylation and ciliary beating observed in response to a standardized, flavorless base under acute conditions highlight the biological relevance and importance of our findings.

## Conclusions

In this study, we show that e-cig exposure induces a heterogeneous response in ALI cultures from different donors. Moreover, we show that different flavors lead to varied effects on those cultures. As a result of these varied effects, we moved forward by using unflavored e-cigs composed of PG:VG (30:70 ratio) with 5% nicotine for all subsequent experiments. We hypothesized that those components which are shared by all e-cigs will give us insights and results that can be applicable to other e-cigs that contain flavors as well. Next, we observed a functional change in the ciliated cells by way of slower CBF following unflavored e-cig exposure compared to room air control. This functional change was accompanied by abnormalities in ciliary ultrastructure as confirmed by TEM images. These changes could not be explained by changes in RNA or protein levels; however, we saw significant changes in PTMs, specifically phosphorylation, following e-cig exposure. Further analysis of the phosphoproteomics data revealed the involvement of actin and cadherin binding along with Rho GTPase signaling pathway, all of which have been shown to be involved in ciliogenesis, and cilia structural anchoring. These findings suggest that unflavored e-cig exposure may disrupt ciliary function by altering the phosphorylation state of proteins involved in actin and cadherin binding, as well as Rho GTPase signaling. This disruption could lead to the observed abnormalities in ciliary ultrastructure and function, potentially contributing to dysregulated MCC and the adverse respiratory effects associated with e-cig use.

## Supplementary Information

Supplementary Material 1: Supplemental Figure 1 is associated with Figure 1. Figure 1S: Flavor-to-flavor heterogeneity and donor-to-donor heterogeneity in response to flavors is evident in ALI cultures. (A) Quantification of number of Keratin 5+ in each donor separately across the six exposures. (B) Quantification of number of Ac-Tub+ in each donor separately across the six exposures. (C) Quantification of number of Muc5AC+ mucus cells in each donor separately across the six exposures. (D) Quantification of number of Keratin 5+ in response to each flavor across the three donors. (E) Quantification of number of Ac-Tub+ in response to each flavor across the three donors. (F) Quantification of number of Muc5AC+ mucus cells in response to each flavor across the three donors. Graphs represent mean ± SEM, *n* = 3 biological replicates (3 different donors), each with 3 technical replicates (3 ALI transwell cultures derived from each of the 3 different donors). Each dot represents a replicate. P values are calculated from all technical replicates across the biological replicates.

Supplementary Material 2: Supplemental Figure 2 is associated with Figure 3. Figure 2S: Ciliary beat frequency, differential gene expression and quality control analyses of phosphoproteomics data after exposure to unflavored e-cig aerosols. (A) Percentage ciliary movement in ALI cultures from donor 1 (cyan) and donor 2 (magenta) after unflavored e-cig exposure or room air control. (B) Ciliary beat frequency in ALI cultures from donor 1 (cyan) and donor 2 (magenta) after unflavored e-cig exposure or room air control. (C) List of differential gene expression after unflavored e-cig exposure as compared to room air control. (*n*=2 biological replicates). (D) Gene ontology terms related to molecular function after unflavored e-cig exposure as compared to room air control. (E) Table of proteins that were changed in abundance by exposure to unflavored e-cigs. (n=2 biological replicates). (F) Principal component analysis (PCA) for each replicate of air and unflavored e-cig conditions. (G) Number of total detected phosphorylated and non-phosphorylated peptides from the phosphoproteomics analysis in each replicate for air and unflavored e-cig conditions. (G) graph showing phosphopeptide enrichment efficiency in each sample. (*n*=1 biological replicate, 3 technical replicates)

Supplementary Material 3: Supplementary Table 1. Fold changes and P values for abundance proteomics, and phosphoproteomics datasets.

Supplementary Material 4: Supplementary Table 2. Pathway enrichment results of phosphoproteomics datasets. (i.e. Figure 3 E-G).

Supplementary Material 5: Supplemental Video 1. Shows cilia motility with room air exposure.

Supplementary Material 6: Supplemental Video 2. Shows cilia motility after PG:VG (30:70 ratio) with 5% nicotine exposure.

## Data Availability

The mass spectrometry proteomics data have been deposited to the ProteomXchange Consortium via the Proteomics Identification Database (PRIDE) partner repository with the dataset identifier PXD059015 Reviewer access details: Login to the PRIDE website using the following details: Project accession: PXD059015 Token: mXXMlB07FamL Alternatively, reviewers can access the dataset by logging in to the PRIDE website using the following account details: Username: reviewer_pxd059015@ebi.ac.uk Password: ElA9enLeGHwc. Reviewer access details: Login to the PRIDE website using the following details: Project accession: PXD059015. Token: mXXMlB07FamL. Alternatively, reviewers can access the dataset by logging in to the PRIDE website using the following account details: Username: reviewer_pxd059015@ebi.ac.uk. Password: ElA9enLeGHwc.

## Abbreviations

VG: Vegetable glycerin
PG: Propylene glycol
ALI: Air–liquid interface
MCC: Mucociliary clearance
COPD: Chronic Obstructive Pulmonary Disease
IPF: Idiopathic pulmonary fibrosis
e-cigs: E-cigarettes
CBF: Cilia beat frequency
PCD: Primary ciliary dyskinesia
PTMs: Post-translational modifications
IIAM: International Institute for the Advancement of Medicine
ABSC: Airway basal stem cell
FOVs: Fields of view
TBST: Tris-Buffered Saline and Tween-20
TEM: Transmission Electron Microscope
KRT5: Keratin 5
Muc5AC: Mucin 5AC
Ac-Tub: Acetylated-alpha tubulin
GO: Gene ontology
gomf: GO Molecular Function
STRING: Search Tool for the Retrieval of Interacting Genes/Proteins
IRB: Institutional Review Board
PRIDE: Proteomics Identification Database
