# Supplementary figures and images for: Unflavored electronic cigarette exposure induces alterations in airway ciliary structure and function
Source: Respir Res. 2025 Jul 2;26:223. doi: 10.1186/s12931-025-03302-w (PMC12224850; doi:10.1186/s12931-025-03302-w)

**A**

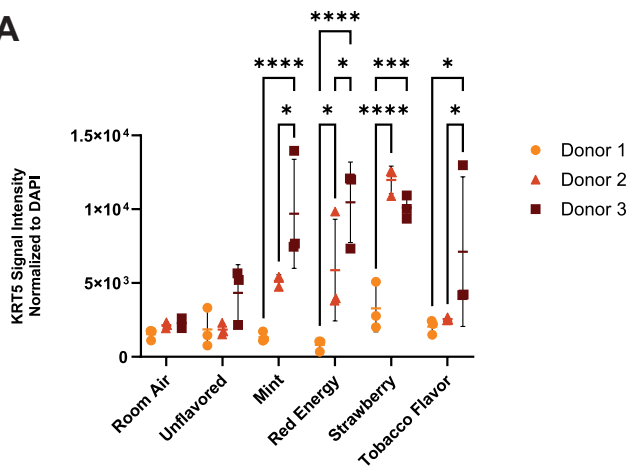

# B

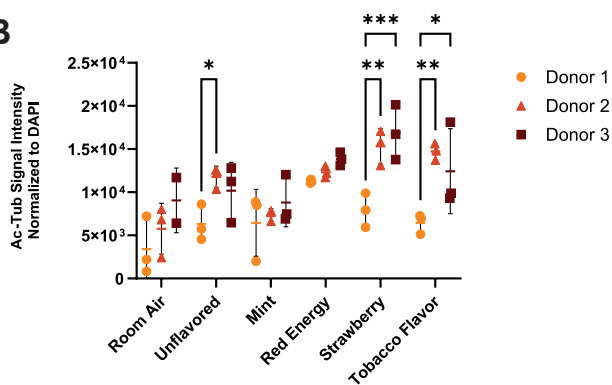

**C**

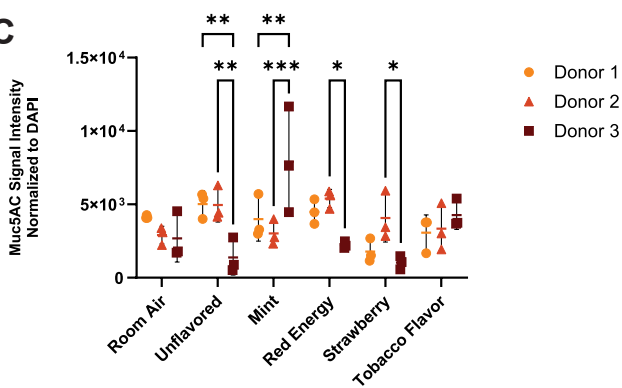

## D

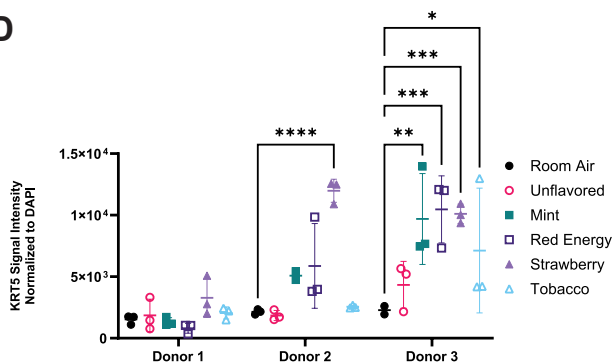

## E

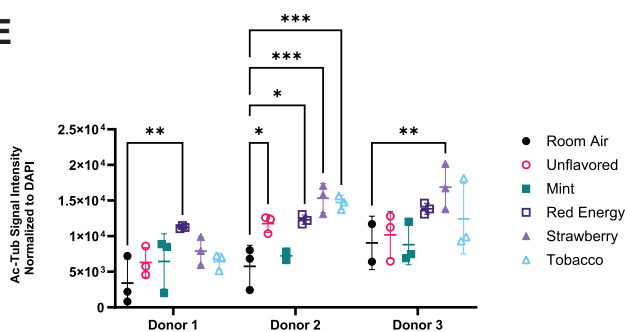

## F

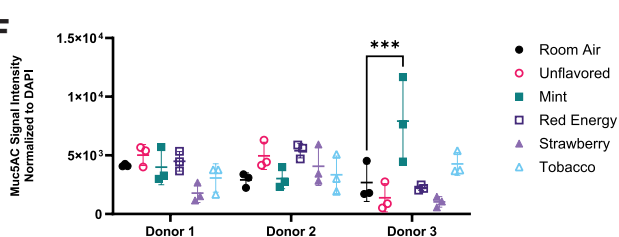

Supplement: Supplementary file 1 — Supplementary Material 1: Supplemental Figure 1 is associated with Figure 1. Figure 1S: Flavor-to-flavor heterogeneity and donor-to-donor heterogeneity in response to flavors is evident in ALI cultures. (A) Quantification of number of Keratin 5+ in each donor separately across the six exposures. (B) Quantification of number of Ac-Tub+ in each donor separately across the six exposures. (C) Quantification of number of Muc5AC+ mucus cells in each donor separately across the six exposures. (D) Quantification of number of Keratin 5+ in response to each flavor across the three donors. (E) Quantification of number of Ac-Tub+ in response to each flavor across the three donors. (F) Quantification of number of Muc5AC+ mucus cells in response to each flavor across the three donors. Graphs represent mean ± SEM, n = 3 biological replicates (3 different donors), each with 3 technical replicates (3 ALI transwell cultures derived from each of the 3 different donors). Each dot represents a replicate. P values are calculated from all technical replicates across the biological replicates. [file 12931_2025_3302_MOESM1_ESM.pdf]

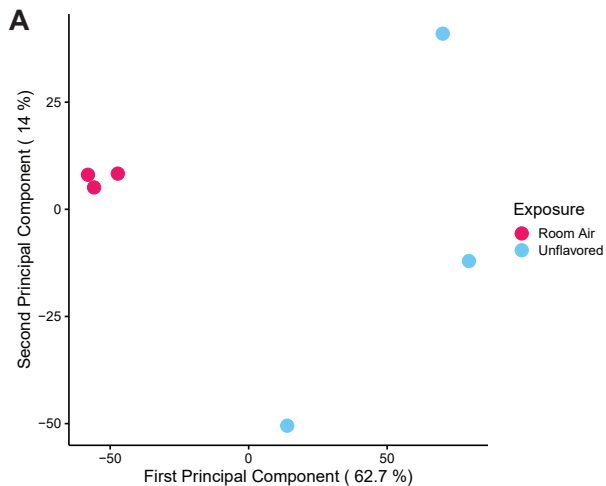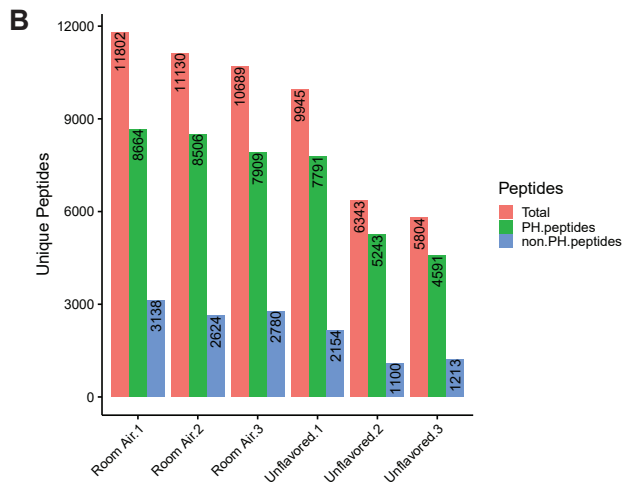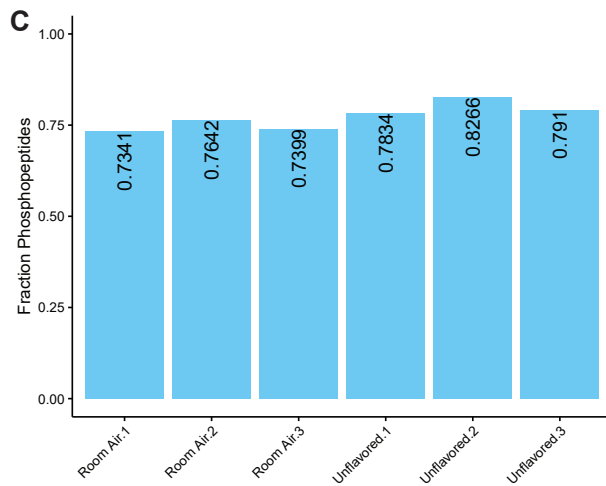

Supplement: Supplementary file 2 — Supplementary Material 2: Supplemental Figure 2 is associated with Figure 3. Figure 2S: Ciliary beat frequency, differential gene expression and quality control analyses of phosphoproteomics data after exposure to unflavored e-cig aerosols. (A) Percentage ciliary movement in ALI cultures from donor 1 (cyan) and donor 2 (magenta) after unflavored e-cig exposure or room air control. (B) Ciliary beat frequency in ALI cultures from donor 1 (cyan) and donor 2 (magenta) after unflavored e-cig exposure or room air control. (C) List of differential gene expression after unflavored e-cig exposure as compared to room air control. (n=2 biological replicates). (D) Gene ontology terms related to molecular function after unflavored e-cig exposure as compared to room air control. (E) Table of proteins that were changed in abundance by exposure to unflavored e-cigs. (n=2 biological replicates). (F) Principal component analysis (PCA) for each replicate of air and unflavored e-cig conditions. (G) Number of total detected phosphorylated and non-phosphorylated peptides from the phosphoproteomics analysis in each replicate for air and unflavored e-cig conditions. (G) graph showing phosphopeptide enrichment efficiency in each sample. (n=1 biological replicate, 3 technical replicates) [file 12931_2025_3302_MOESM2_ESM.pdf]
